# Synthesis of Nucleoside Analogs Containing Sulfur or Selenium Replacements of the Ribose Ring Oxygen or Carbon

**DOI:** 10.1021/acs.joc.4c02409

**Published:** 2024-11-21

**Authors:** Caecilie M. M. Benckendorff, Peter Sunde-Brown, Aisling Ní Cheallaigh, Gavin J. Miller

**Affiliations:** School of Chemical and Physical Sciences and Centre for Glycoscience, Keele University, Keele, Staffordshire ST5 5BG, United Kingdom

## Abstract

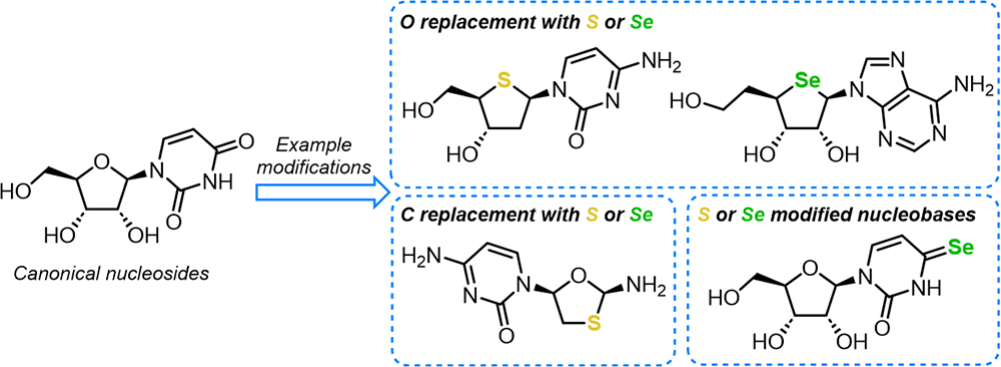

Nucleoside analogs have proven highly successful in many pharmaceutical intervention strategies, and continued exploration of next generation structural motifs is required. Herein we discuss recent advances toward the chemical synthesis of heteroatom-modified nucleosides, where this is constituted by the chalcogens sulfur or selenium. Paying specific focus to the organic chemistry to incorporate these heteroatoms, we consider developments toward ribose ring oxygen and ring carbon replacements alongside chalcogen-modified heterobases.

Canonical nucleosides constitute the building blocks for the biomolecules of life. They are integral components of oligonucleotides (DNA and RNA) that encode the proteins required to control biological processes. Nucleosides are also omnipresent within enzyme cofactors (NAD^+^/H), as cellular energy sources (ATP) and as secondary signaling molecules (cyclic dinucleotides). Synthetic analogs of nucleosides and nucleotides therefore represent a successful strategy to develop therapeutics that target diverse biological processes. These include DNA replication, transcription, translation and cell signaling. To exemplify, nucleoside analogs possess a privileged and accomplished history within therapeutic intervention strategies, most notably against viruses and cancer^1,2^ and recently as frontline treatments against COVID-19.^3,4^

Nucleoside and nucleotide analog therapeutics may contain chemical modifications to component parts of the d-ribose sugar, the heterobase and 5′-*O*-phosphate, which confer their favorable pharmacological profile(s). One such modification is the replacement of canonical oxygen and carbon atoms within d-ribose with another chalcogen (herein sulfur or selenium). Chalcogen switching changes the hydrolytic stability and/or conformational plasticity of the derived ring, potentially offering an alternative or improved metabolic profile.

Sulfur and selenium containing nucleosides also occur naturally, exemplified by 2-thiouridine (s^2^U, Figure 1), which is found within derived nucleic acids, highly conserved across all organisms and playing a critical role within tRNA.^5^ 2-Thiolation of uridine confers a more rigid ^3^T_2_ (North) conformation, which effects neighboring nucleotides and reinforces a favored geometry in ssRNA.^6^ These benefits allow for both a higher degree of efficiency within codon translation and improve its reliability.^7^ Furthermore, chalcogen switching at this position with selenium and incorporation of a 5-methyl aminomethyl group gives mnm^5^Se^2^U (Figure 1),^8^ also conserved in derived nucleic acids for a variety of bacteria and archaea.^9,10^

**Figure 1:**
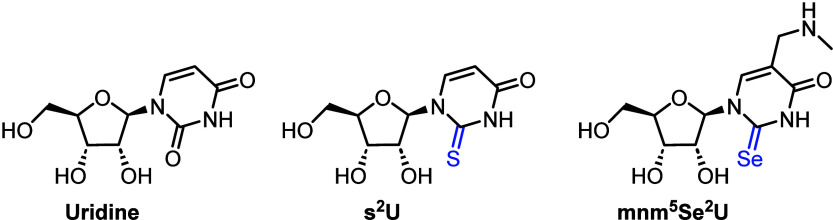
Structure of uridine and the naturally occurring, sulfur- and selenium-containing nucleosides 2-thiouridine (s^2^U) and 5-methylaminomethyl-2-selenouridine (mnm^5^Se^2^U).

This Synopsis will highlight advances toward the synthesis of chalcogen-modified nucleosides over the past ten years. We begin with approaches to incorporate sulfur and selenium into the pentose ring, in place of furanosyl 4-position oxygen. This is followed by methods to incorporate these heteroatoms at other ring positions, replacing carbon, and within the heterobase. We focus on the organic chemistry used to incorporate the chalcogen, followed by a short summary of relevant derived biological activity.

## 1 4-*O*-Ribose Oxygen Replacement with Sulfur

**Figure 2:**
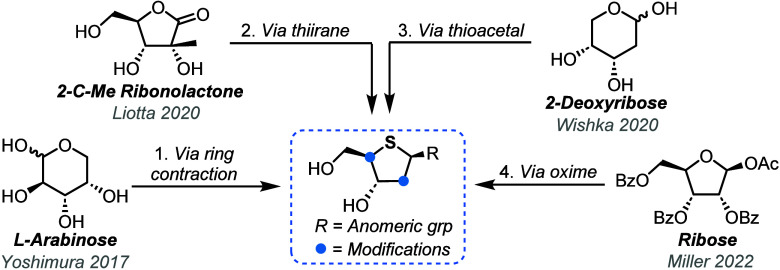
Overview of recent approaches for the synthesis of 4-thioribose scaffolds from commercially available chiral pool materials.

4′-Thionucleosides were conceptualized in the 1960s with a first reported synthesis of 2′-deoxy-4′-thioadenosine by Reist *et al*. in 1964.^11^ Since then, significant academic and industrial research efforts have broadened the number of compounds containing this modification, toward analogs for pharmaceutical development.^12−17^ Although the replacement of the sugar ring oxygen with sulfur has been shown to impart beneficial pharmacokinetic properties,^18−23^ there are currently no approved 4′-thionucleoside therapeutics. Offering solutions to alleviate a synthetic bottleneck for the development of further generations of 4′-thionucleoside analogs, several groups have now developed scalable and efficient syntheses of requisite 4-thiosugar building blocks (Figure 2).

### 1.1 A Ring Contraction–Expansion–Contraction Approach

**Scheme 1:**
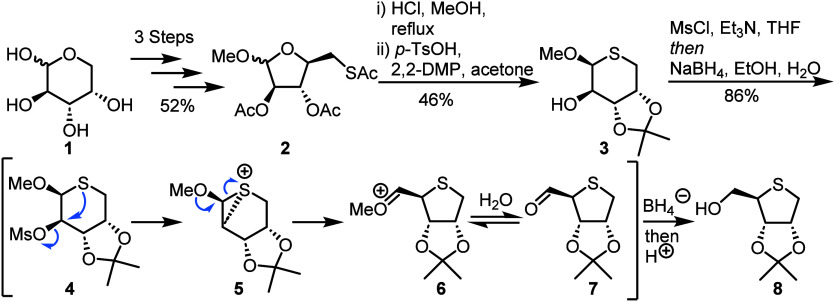
Synthesis of 4-Thioribitol **8** from l-Arabinose Using a Ring Contraction/Reduction Cascade Sulfur insertion *via* C5 thioacetate installation, followed by acidic deacetylation, enables cyclisation to 5-thiopentose **3**. Ring contraction is then facilitated through a reductive episulfonium rearrangement.

Yoshimura and co-workers reported a practical synthesis of 4′-thiopyrimidine nucleosides, describing a novel ring-contraction protocol to prepare a 4-thioribitol intermediate **8** (Scheme 1), followed by Pummerer-type glycosidation of *N*^4^-Ac cytosine and uracil using this material.^24^ Starting from commercially available l-arabinose **1**, intermediate methyl glycoside **2** was obtained in 52% yield over three steps. Next, an acidic deacetylation facilitated ring expansion and subsequent 3,4-*O*-isopropylidene protection afforded thiosugar **3** in 46% yield over two steps. A second ring contraction was achieved *via* an episulfonium rearrangement (Scheme 1, indicative mechanism *via* intermediates **4**-**7**) under reductive conditions to furnish 4-thioribitol **8** in 86% yield. The synthesis was completed on multigram scale, delivering **8** in 21% yield over seven steps.

### 1.2 Ring Opening a C4–C5 Thiirane

**Scheme 2:**
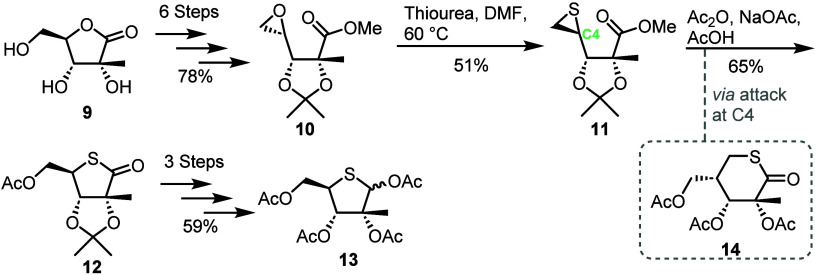
Synthesis of 2-*C*-Methyl-4-thioribose **13** from Commercial 2-*C*-Methyl Ribonolactone **9** Stereoinversion and sulfur insertion is performed with thiourea. Subsequent thiirane ring opening is accomplished following nucleophilic attack by acetate, facilitating ring closure to thiolactone **12**. Unwanted nucleophilic attack at C4 was also observed (grey dotted box).

Liotta and co-workers reported the synthesis of six 2′-*C*-methyl-4′-thionucleoside analogs along with their activity against Hepatitis C virus (HCV).^25^ Based on the work of Dukhan *et al*.,^26^ the group synthesized a 4-thioribose scaffold from commercial 2-*C*-methyl-ribonolactone **9**, converting to l-*lyxo*-configured epoxide intermediate **10** in 78% yield over six steps (Scheme 2). Sulfur insertion and stereoinversion was then achieved through treatment of epoxide **10** with thiourea, furnishing thiirane **11** in 51% yield. Acidic thiirane opening and cyclization to 4-thiolactone **12** was then accomplished in 65% yield. Optimisation of this step was necessary to minimize the formation of an undesired 6-membered thiolactone **14**, a byproduct of nucleophilic attack at C4 (Scheme 2, gray dotted box). Lastly, following reduction of lactone **12**, deprotection and global acetylation, 2-*C*-methyl-4-thioribose **13** was obtained in 59% over three steps. The synthesis was completed starting from 5 g of lactone **9**, accessing 4-thioribose derivative **13** in 15% yield over 11 steps.

### 1.3 Exploiting an Aldehyde Protecting Group - Thioacetals

**Scheme 3:**
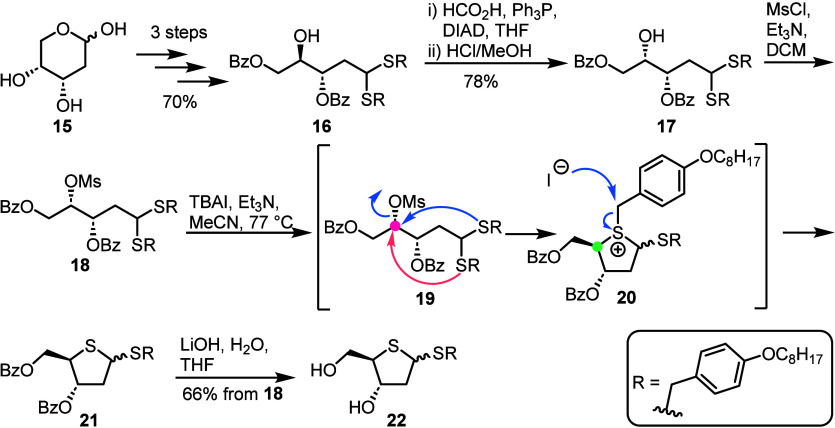
Scalable Synthesis of 4-Thioribo Thioglycoside **22** from 2-Deoxyribose **15** Following stereoinversion at C4 and conversion to mesylate **18**, cyclisation by nucleophilic attack from the dithioacetal resulted in an anomeric thioglycoside mixture.

In 2014, Thottassery *et al*. identified two 4′-thionucleoside analogs, 2′-deoxy-4′-thiocytidine (TdCyd) and 5-aza-2′-deoxy-4′-thiocytidine (Aza-TdCyd) as potent DNA methyltransferase I (DNMT1) inhibitors.^27^ Recognizing the potential pharmaceutical importance of these analogs, Wishka and co-workers revised and optimized previous syntheses to facilitate multigram scale production.^28^ Building a route to similar thioglycoside donors,^13^ scalable synthesis of key thioglycoside **22** was achieved using two separate routes. One of these, starting from 2-deoxyribose **15** is highlighted in Scheme 3 and furnished 43 g of 2-deoxy glycoside **22** in 36% yield over eight steps.

Sulfur insertion was achieved following a series of protecting group manipulations from 2-deoxyribose **15** and open-chain form locking as dithioacetal intermediate **16** in 70% yield over three steps. Stereoinversion at C4 was performed using Mitsunobu conditions with a formate nucleophile, which was subsequently cleaved to furnish free C4-alcohol **17** in 78% over two steps. Stereoinversion at C4 was essential for overall retention of the d-*ribo* configuration upon conversion to thiosugar **22**. Next, ring closure was enabled through installation of a mesylate at C4 to give open-chain dithioacetal **18**, followed by treatment with TBAI and Et_3_N to give protected thioglycoside **21**. This process is indicated to proceed *via* sulfonium **20**, then subject to iodide-mediated dealkylation.^29^ Free thioglycoside **22** was thence obtained after saponification of compound **21**, using LiOH, as a mixture of anomers in 66% yield over two steps. The synthesis of thioglycoside **22** was achieved over eight steps, starting from 121 g of **15**. It should be noted that the synthesis described required a parallel synthesis of the thiol necessary to form dithioacetal **16**, achieved on kilogram scale in 79% yield over two steps.

More recently, Morris *et al*. demonstrated the practicality of this method on a commercially available 2-fluoro-modified ribose scaffold, facilitating kilogram-scale production of a 2-deoxy-2-β-fluoro-4-thioribose building block,^30^ requiring minor modification to the synthesis highlighted in Scheme 3.

### 1.4 Exploiting an Aldehyde Protecting Group: Oximes

**Scheme 4:**
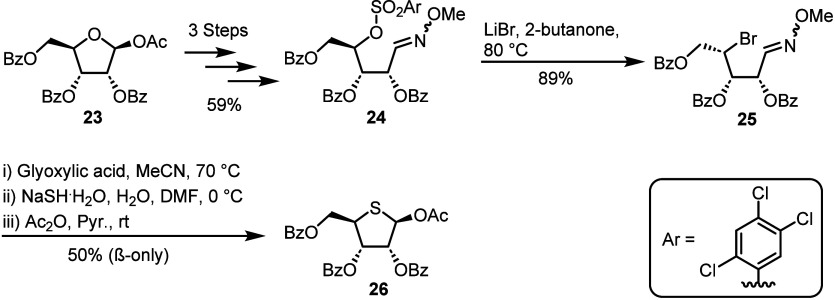
Synthesis of 4-Thioribose Scaffold **26** from Commercial **23** Stereoinversion at C4 was performed *via* sulfonate displacement with bromide. Hydrolysis of the oxime with glyoxylic acid and subsequent treatment with NaSH facilitated cyclisation. Isolation of the β-anomer was completed following anomeric acetylation, to furnish **26**.

Miller *et al*. demonstrated a scalable and chromatography free synthesis of protected 4-thioribose **26** from commercially available acetate **23** (Scheme 4).^31^ Key to maintaining the d-*ribo* configuration was a double inversion at C4. First, S_N_2 displacement of an aryl sulfonyl leaving group with bromide afforded l-*lyxo* configured bromide **25** in 89% yield. Importantly, the choice of 2-butanone solvent proved critical, as other solvents formed mixtures of C4 diastereoisomers. A second S_N_2 using NaSH regenerated a d-*ribo* configuration and ring closure was achieved following release of the C1 aldehyde with glyoxylic acid, granting access to gram quantities of 4-thioribose **26**, isolated as the β-anomer in 50% yield over three steps. This chromatography-free synthesis was delivered over seven steps in 26% yield from 100 g of **23**. The practicability of the method was mapped onto 2-deoxy-2-fluoro-2-*C*-methyl and 2-deoxy-2-*gem*difluorinated ribonolactone scaffolds, granting access to several 2′-modified 4′-thioribo nucleosides.^31,32^

### 1.5 Summary and Indicative Biological Activity

**Figure 3:**
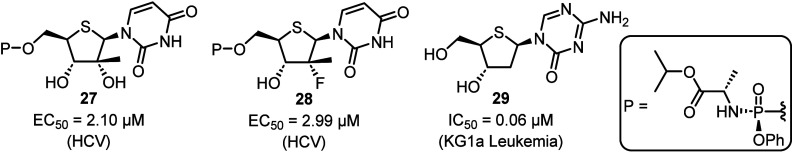
Structures and biological activities of 4′-thionucleoside analogs **27**,^25^**28**^32^ and **29**,^28^ accessed from 4-thiofuranose building blocks.

Advances in the development of robust and scalable syntheses of 4-thiofuranosyl building blocks have yielded important progress (*vide supra*), facilitating access to derived 4′-thionucleosides. Utilizing these important intermediates, Liotta and co-workers synthesized a 2′-*C*-methyl-4′-thiouridine prodrug **27** (Figure 3),^25^ which exhibited potent activity against HCV (EC_50_ = 2.10 μM). Similarly, Miller *et al*. reported HCV activity for the 4′-thio analog of sofosbuvir, **28** (EC_50_ = 2.99 μM).^32^ Although an approximate 100-fold reduction in potency was observed compared to sofosbuvir (EC_50_ = 0.05 μM), both analogs displayed no significant cytotoxicity at high concentrations (**27** and **28**: CC_50_ > 100 μM; sofosbuvir: CC_50_ > 5 μM). These findings suggest that while bioisosteric replacement of oxygen with sulfur may reduce potency, there is an associated reduction in toxicity. 2′-Deoxy-4′-thiocytidine **29**, reported by Wishka and colleagues, demonstrated anticancer activity (IC_50_ = 0.06 μM against KG1a leukemia cells), and is currently undergoing clinical trials.

## 2 4-*O*-Ribose Oxygen Replacement with Selenium

**Figure 4:**
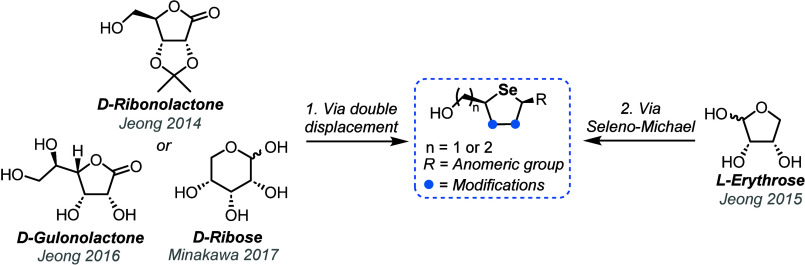
Overview of approaches for the synthesis of 4-selenosugar scaffolds from commercial chiral pool starting materials.

4′-Selenonucleosides were established in 2007, with a first synthesis reported by Jeong and co-workers.^33^ Over the 17 years since, the general synthetic approach has remained largely consistent, typically attempted in two ways (Figure 4). The first uses a double displacement of suitable open chain leaving groups with a selenium nucleophile and the second more recent chemistry uses a seleno-Michael addition.

### 2.1 Double Nucleophilic Substitution Approach

**Scheme 5:**
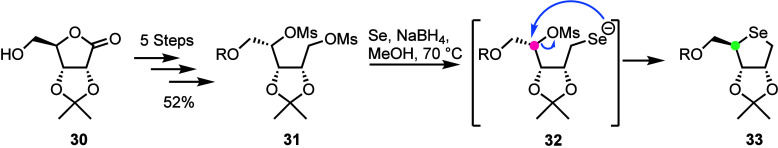
Synthesis of 4-Selenoribitol **33** from Commercial d-Ribonolactone **30** Selenium insertion was achieved *via* a double inversion, first displacing primary C1 mesylate followed by cyclisation and stereoinversion at C4. R= TBDPS.

The synthesis of a key 4-selenoribitol intermediate **33** was described by Jeong and co-workers in 2014, utilizing commercially available d-ribonolactone **30** (Scheme 5).^34,35^ The initial conversion to l-lyxo-configured bismesylate **31** was accomplished through a five-step process in 52% yield on gram scale. Following this, selenium insertion was completed via double displacement of the mesylate leaving groups. The selenium was reduced *in situ* with NaBH_4_ to generate the reactive species, NaHSe, which facilitated displacement of the primary C1-mesylate. Subsequently, cyclization and C4 stereoinversion to the desired d-*ribo* configuration was facilitated through nucleophilic displacement by C1-selenide intermediate **32**. This synthetic approach was later mapped onto a 2-*C*-methyl-ribonolactone scaffold, granting access to 2′-*C*-methyl-4′-selenonucleosides.^36,37^

**Scheme 6:**
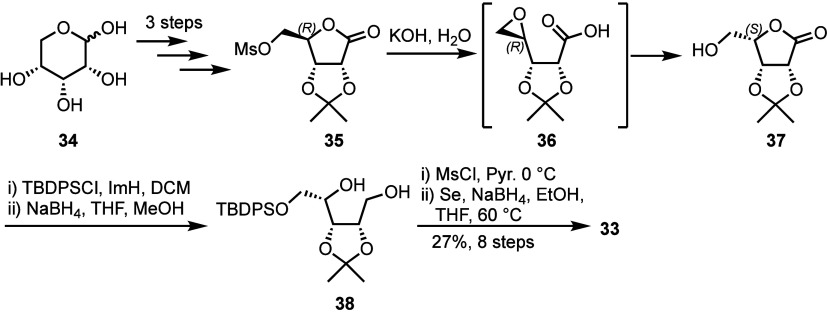
Synthesis of 4-Selenoribitol **33** from d-Ribose **34** Selenium insertion is performed *via* double displacement, as illustraated previously.

Minakawa and colleagues also reported a scalable and practical synthesis of 4-selenoribitol **33** from d-ribose (Scheme 6).^38^ Starting from 45 g of d-ribose **34** and telescoping the entire synthesis over eight steps, purifying only after the last synthetic step, afforded protected selenoribitol **33** in 27% yield.^38^ The key stereoinversion at C4 was achieved using lactone opening with hydroxide followed by intramolecular displacement within mesylate **35** to form epoxide **36**, which was then ring opened by the terminal carboxylic acid to furnish lactone **37**. Notably, key intermediates in this synthetic route are commercially available, including l-lyxonolactone **37**, making this an attractive route for rapid building block access.

### 2.2 Chalcogen Insertion Harnessing a Seleno-Michael Addition

**Scheme 7:**
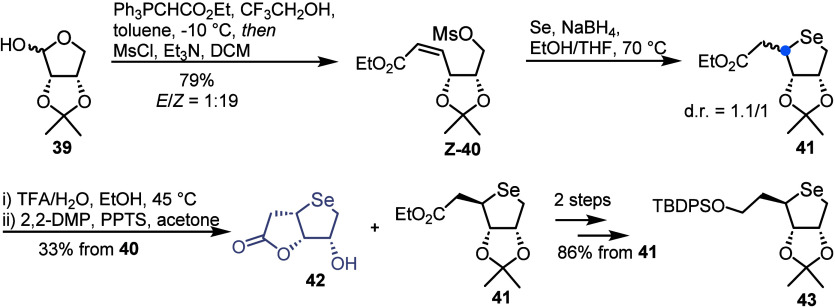
Synthetic Approach to 5-Homo-4-selenoribitol Intermediate Harnessing a Seleno-Michael Reaction for Insertion of the Chalcogen

Jeong and co-workers have also pursued the synthesis of 5′-homo-4′-selenonucleosides using a novel seleno-Michael addition (Scheme 7).^39^ Starting from l-erythrofuranose derivative **39**, treatment with Wittig reagent Ph_3_PCHCO_2_Et in the presence of trifluoroethanol, followed by mesylation furnished alkene *Z*-**40** (*E*/*Z* = 1:19). The authors did not comment on the unusual geometric outcome of the Wittig reaction, favoring *Z*-**40** over the expected *E*-**40** when using a stabilized ylide. Next, a seleno-Michael addition was achieved through treatment of alkene **40** with selenium powder and NaBH_4_, affording 5-homo-4-selenoribitol **41** as an 1.1/1 mixture (d-*ribo*/l-*lyxo*). To aid the isolation of the desired d-**41**, the mixture was subjected to isopropylidene hydrolysis, where the l-*lyxo* diastereoisomer formed cyclic lactone **42**. Following isopropylidene reprotection, d-*ribo*-**41** was isolated in 33% yield from *Z*-**40***via* column chromatography. 5-Homo-4-selenoribitol intermediate **43** was then obtained following reduction of the ester and silyl protection.

**Scheme 8:**
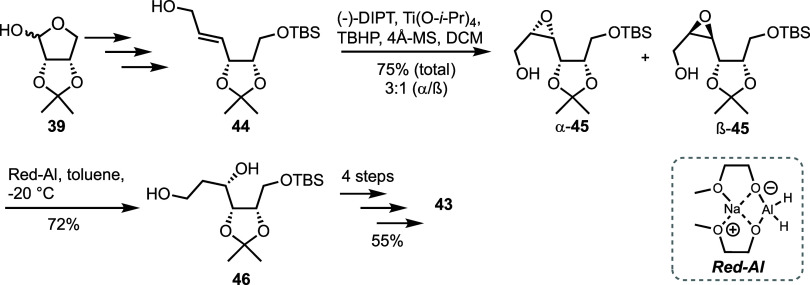
Alternative Approach to the Synthesis of 5-Homo-4-selenoribitol **43** from l-Erythrose **39** Utilising an Asymmetric Sharpless Epoxidation Followed by Regioselective Reductive Ring Opening

To improve the synthesis of 5-homo-4-selenoribotiol derivative **43**, Jeong and co-workers sought to eliminate formation of the undesired l-*lyxo* isomer during selenium insertion by utilizing Sharpless asymmetric epoxidation followed by regioselective opening of the resultant epoxide (Scheme 8).^40^ The authors improved the yield for this key transformation to 75% and diastereoselectivity to 3:1 in favor of the desired α-**45** configuration. While the α/β epoxide mixture was inseparable, upon reductive ring opening the resultant mixture of primary alcohols was separable *via* column chromatography, and diol **46** was obtained in 72% yield. Selenium insertion was then performed following the double displacement methodology, furnishing **43** in 55% over four steps.

**Scheme 9:**
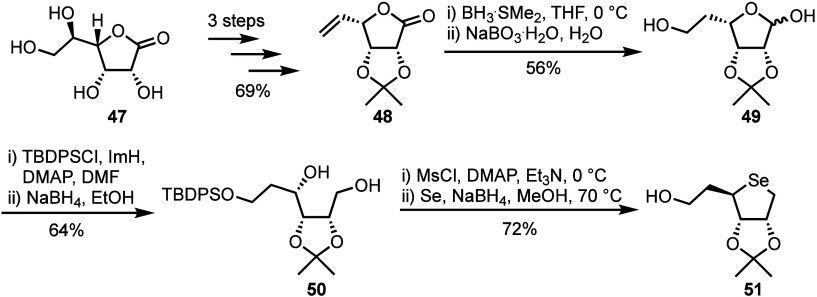
Key Synthetic Steps in the Synthesis of 5-Homo-4-selenoribitol **51** from d-Gulonic γ-Lactone, Avoiding Diastereoisomer Separation C5-homologation was achieved *via* a hydroboration-oxidation of l-lyxonolactone intermediate **48**. Following primary hydroxyl protection and lactol reduction, selenium insertion was performed *via* double displacement.

Further addressing the challenges posed by unwanted diastereomer formation, Jeong and co-workers have more recently introduced a revised synthetic strategy to reach selenoribitol **51**, starting from d-gulonic γ-lactone **47** (Scheme 9).^41^ Following conversion to 5,6-exo alkene **48** using a previously established procedure,^42^ treatment with NaBO_3_•H_2_O afforded primary alcohol **49** in 56% yield. The primary alcohol was silyl protected, and the resultant lactol reduced with NaBH_4_ to furnish diol **50** in 64% yield. From here, selenium insertion was achieved via double displacement as described previously, in 72% yield.

### 2.3 Summary and Indicative Biological Activity

Given the relatively recent introduction of 4′-selenonucleoside analogs, it is unsurprising that fewer synthetic methods have been developed. To synthesize 4′-selenonucleosides from either 4-selenoribitol **33** or 5-homo-4-selenoribitol **43**, the selenium atom is first oxidized to the corresponding selenoxide.^33,43^ Following oxidation, glycosylation to a nucleobase is achieved through either a Pummerer-type glycosylation (for pyrimidines) or by first performing anomeric acetylation (*via* a Pummerer reaction with an acetate nucleophile) followed by glycosylation under Vorbrüggen conditions. To avoid the need for glycosylation using Vorbrüggen conditions when working with purine nucleobases, Minakawa *et al*. described a method using a hypervalent iodine oxidant (iodosobenzene) in combination with TMSOTf.^44^ Although this method was effective, it resulted in the formation of undesired *N*^7^-regioisomers.

**Figure 5:**
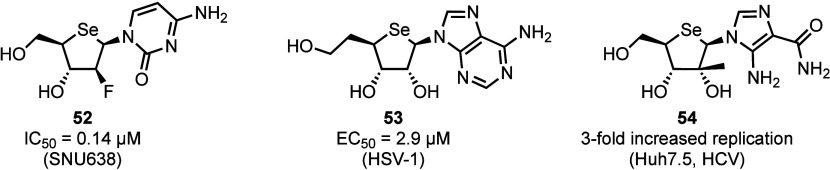
Structures and biological activities of three 4′-selenonucleoside analogs.

In general, there are fewer reports of biological activity for 4′-selenonucleosides compared to their 4′-oxo and 4′-thio counterparts. Noteworthy among the data reported is 2′-deoxy*-*2′*-*fluoro-4′-seleno-arabinocytidine **52** (Figure 5), which demonstrated anticancer activity (IC_50_ = 0.14–1 μM),^35,45^ surpassing the control drug cytarabine. Interestingly, no phosphorylated forms of selenonucleoside **52** were detected using LCMS analysis.^39^ Furthermore, 5′-homo-4′-selenoadenosine **53** showed activity against HSV-1 (EC_50_ = 2.9 μM),^39,46^ and in contrast to analog **52**, 6′-*O*-phosphorylation was successfully detected.^40,47^ This was hypothesized to be enabled through a greater distance between the 6′-hydroxyl and the bulkier selenium atom, reducing steric hindrance for phosphorylation by cellular kinases. In an unexpected development, a 2′-*C*-methyl-4′-seleno derivative of the pharmaceutical agent acadesine, **54**, was found to promote HCV RNA replication 3-fold. The cause of this effect has not been identified.^48^

## 3 Nucleoside Analog Ring Carbon Replacement with a Chalcogen

**Figure 6:**
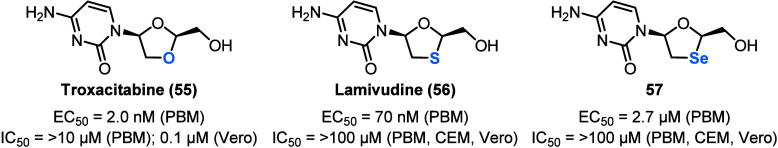
Comparison of the biological activity of nucleoside analogs bearing a C3′-replacement of carbon with oxygen (**55**), sulfur (**56**) and selenium (**57**). Lamivudine **56** is used for the treatment of HIV-1 and HBV.

Increasing structural diversity within the sugar ring has proven critical to the development of nucleoside analog therapeutics. Alternate structural flexibility can be afforded through the introduction of chalcogens in d-ribose ring positions other than that of native oxygen. In addition to alternate conformational plasticity, the additional chalcogen may enable altered hydrogen bonding interactions. Intricate differences in therapeutic activity can be observed as the size of the heteroatom increases (O vs S vs Se), exemplified in Figure 6 by comparing the anti-HIV-1 activity of the dioxalane therapeutic troxacitabine (**55**), the oxathiolane therapeutic lamivudine (**56**) and oxaselenolane analog (**57**).^48,49^ Troxacitabine **56** displays excellent antiviral activity, however it is also cytotoxic in several cell lines.^49^ When the additional C3′ ring oxygen is changed to sulfur or selenium, no cytotoxicity is observed, while antiviral activity is retained.^48^ It is possible that human DNA polymerase is able to tolerate the smaller oxygen analog **55**, leading to cytotoxicity, while the bulkier analogs **56** and **57** are not accepted, but are utilized by promiscuous viral transcriptases.

### 3.1 C3-Chalcogen Nucleosides Using a Chiral Auxiliary Strategy

**Scheme 10:**
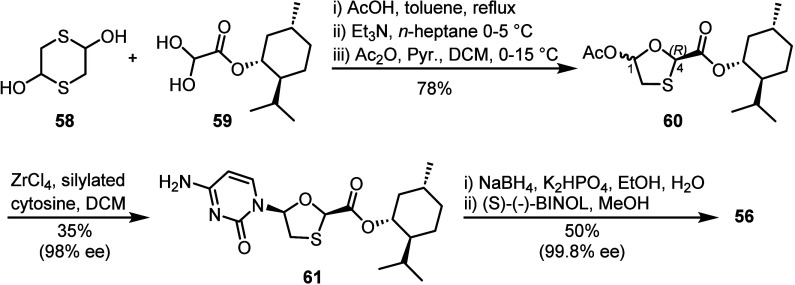
Synthesis of **56** Using a Chiral Auxiliary Strategy The core oxathiolane is formed by reaction of dithiane diol **58** with L-menthyl glyoxylate **59**. ZrCl_4_-mediated glycosylation delivers analog **61** in 98% ee and is further enriched following reduction and co-crystallization with (S)-(−)-BINOL.

Several reports utilizing L-menthol as a chiral auxiliary to synthesize lamivudine **56** have been disclosed recently.^50−55^ To exemplify, Shenoy and co-workers reported a multikilogram scale preparation from dithiane diol **58** and l-menthyl glyoxylate **59** (Scheme 10).^56^ Stereochemical control at C4 toward the desired *R*-configuration was achieved upon treatment of the oxathiolane product of reaction between **58** and **59** with Et_3_N and subsequent acetylation of the hemiacetal intermediate under careful temperature control in the presence of substoichiometric amounts of pyridine, affording acetate **60** in 78% yield (with 6% of the undesired 4*S* isomer). Next, stereoselective *N*-glycosylation with cytosine was achieved in the presence of ZrCl_4_, furnishing β-nucleoside **61** in 98% ee and 35% yield. Further enantiomeric enrichment was achieved after cleavage of the ester and cocrystallization with (*S*)-(−)-BINOL to furnish **56** in 50% yield and 99.8% ee.

### 3.2 C3-Chalcogen Nucleoside Building Blocks Using Biocatalysis

**Scheme 11:**
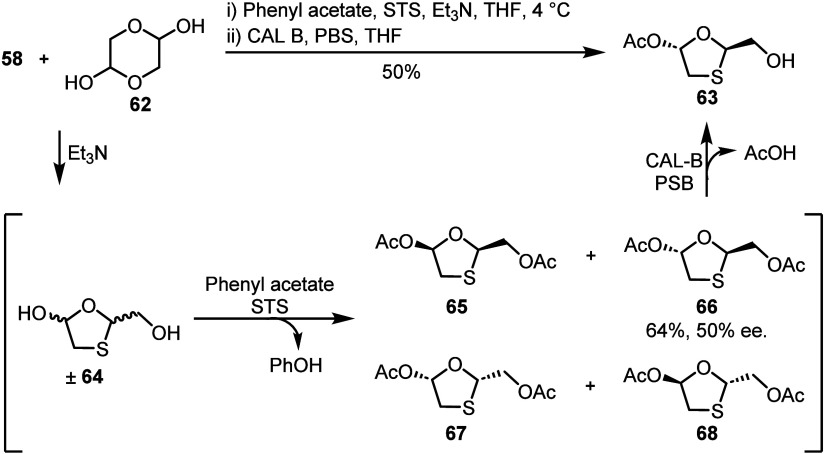
Biocatalytic Synthesis of Key Oxathiolane **63** from Dithiane **58** and Glycolaldehyde Dimer **62** via a Two-Enzyme Cascade

Biocatalytic approaches to the synthesis of lamivudine have also been developed. Building on previous work,^57^ Ramström and co-workers reported a one-pot two-enzyme process to synthesize oxathiolane **63** from dithiane diol **58** and glycolaldehyde dimer **62** (Scheme 11).^58^ Upon formation of racemic oxathiolane **64**, acetylation by surfactant-treated subtilisin Carlsberg (STS) and phenylacetate favored formation of *trans*-diastereoisomer **66** in 50% ee. Upon full consumption of ± **64**, the addition of a second enzyme, *Candida antarctica* lipase B (CAL-B), catalyzed a primary deacetylation of **66**, furnishing **63** exclusively in 50% yield and >99% ee. Although this method was completed only on milligram scale, affording 66 mg of **63**, it demonstrates the potential of enzymatic approaches to solve synthetic bottlenecks. Relatedly, Gao and co-workers utilized whole cell *Klebsiella oxytoca*(59) and Liu and co-workers utilized a *Trichosporon laibachii* lipase^60^ to resolve isomeric mixtures of oxathiolanes in up to 99.9% ee.

### 3.3 Alternative Thionucleoside Analogs

**Scheme 12:**
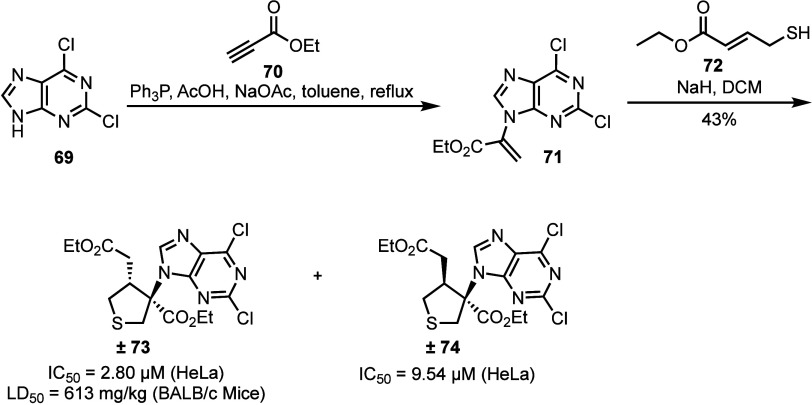
Synthesis of Novel Five-Membered Thionucleoside Analogs *via* a Michael Addition

Chalcogen-modified nucleoside analogs containing a five-membered heterocyclic ring modified beyond that of the 4′-chalcogen or an oxathiolane are rarer, with only a handful of reports between 1990 and 2005.^61−63^ Recently, Guo and co-workers reported a strategy to prepare novel thionucleoside analogs as racemic mixtures using a Michael addition of *trans-*ethyl 4-mercapto-2-butenaoate **72** onto purine bearing acrylate derivative **71**, concomitantly varying the nucleobase substitution pattern (Scheme 12).^64,65^ First, 2,6-dichloropurine **69** was coupled with ethyl propiolate **70** to deliver **71**, at which point reaction with thiol **72** furnished the novel nucleosides ± **73** and ± **74**. A library of twenty-two analogs were synthesized using this approach and therefrom evaluated for anticancer activity. Racemic ± **73** demonstrated inhibitory activity more potent than cisplatin in HeLa cells (IC_50_ = 2.80 μM, ± **73**; IC_50_ = 7.99 μM, cisplatin), and was found to have an LD_50_ of 613 mg/kg *in vivo* in BALB/c mice, lower than the positive controls of 5-fluorouridine (LD_50_ = 230 mg/kg) and cisplatin (LD_50_ = 16.9 mg/kg). These results indicate alternative thionucleoside ± **73** as an attractive scaffold for further development.

## 4 Chalcogen Modification of the Nucleobase

Provision of DNA sequences with major groove modification provides versatile tools for studying related biological chemistry. It also offers capability for developing new diagnostic tools, creating advanced materials, and synthesizing modified nucleic acid sequences. Such changes are often incorporated through modifications at the 5-position of pyrimidine or the 7-position in 7-deazapurines, as these tend to be relatively well tolerated by DNA polymerases.

### 4.1 Chemical Approaches to Install Chalcogen-Modified Nucleobases

**Scheme 13:**
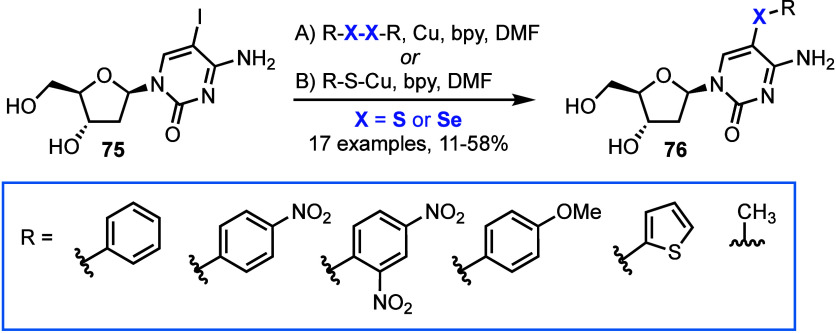
Synthesis of 5-Chacogen Modified 2′-Deoxycytidines, Representative of Chemistry Also Completed for 5-Iodoridine, 7-Deaza-7-iodoadenosine and 7-Deaza-7-iodoguanosine bpy = 2,2′-bipyridyl.

Hocek and co-workers recently reported a copper-mediated 5-position sulfenylation or selenylation of 2′-deoxyuridine, 2′-deoxycytidine and 7-position modification of 7-deaza-2′-deoxyadenosine and 7-deaza-2′-deoxyguanosine (Scheme 13).^66^

Two methods were developed: The first, a copper catalyzed reaction of a disulfide or diselenide with a 5-position halogenated nucleoside **75**, and the second utilizing a pregenerated arylsulfanyl- or arylselenyl-cuprate. Both methods were effective, delivering yields between 11% and 58% of base-modified nucleosides **76**, depending on the sulfur/selenium species. The authors were also able to modify related nucleoside mono- and trisphosphates (NMPs and NTPs), albeit in reduced scope (six examples, 5–45% yield for NMPs; three examples, 7–31% yields for NTPs). The three chalcogen modified NTPs were shown to be substrates for KOD XL DNA polymerase, incorporating thio- or seleno-modified 2′-deoxycytidine analogs into 15-, 19- and 31-mer oligonucleotide sequences in primer extension reactions and successfully affording double stranded DNA with chalcogen-modification in both strands, from PCR amplification.

### 4.2 Harnessing Biocatalysis to Install Chalcogen-Modified Nucleobases

**Scheme 14:**
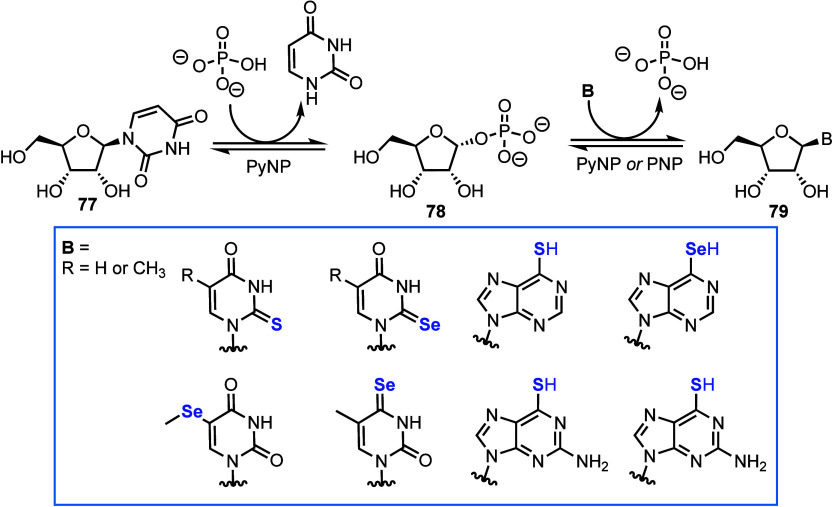
Biocatalytic Transglycosylation of Uridine **76** via Phosphate **78** to Afford Purines and Pyrimidines **79** Containing Chalcogens PyNP = pyrimidine nucleoside phosphorylase; PNP = purine nucleoside phosphorylase.

Huang and co-workers recently reported an enzymatic synthesis of a series of thio- and seleno-nucleosides from uridine or thymidine (Scheme 14).^67^ A one-pot biocatalytic transglycosylation using chalcogen-containing pyrimidine and purine nucleobases from uridine or thymidine was demonstrated, using thermophilic nucleoside phosphorylases (*Tt*PyNP or *Gt*PyNP for pyrimidines and *Tt*PyNP and *Gt*PNP for purines) in moderate to near quantitative conversions (20–97%). Generally, transglycosylation was favored when using uridine **77** as a donor, and higher yielding toward incorporating purines. This seminal work was scaled up in a follow-up report, where milligram quantities of 2-selenouridine, 2-seleno-2′-deoxyuridine, 5-methyl-2-selenouridine and 2-selenothymidine were isolated, following chromatography.^68^

Most recently, a related chemoenzymatic transglycosylation toward natural and halogenated purine and pyrimidine 4′-thionucleosides was demonstrated by Miller and Kurreck, starting from 4′-thiouridine using PyNPs.^69^ A derived biocatalytic synthesis of 5-iodo-4′-thiouridine was scaled up, isolating 70 mg of material, and showcasing the practicability of the method for late-stage diversification of chalcogen-modified nucleoside analogs.

## 5 Summary and Outlook

Chalcogen modification with sulfur and selenium has emerged as an exciting area within the development of synthetic nucleoside analogs. Several methodologies have now been developed for substituting canonical oxygen in the sugar ring with sulfur or selenium, importantly facilitating large-scale synthesis capability for the development of next generation 4′-thio- and 4′-selenonucleosides. While chemical synthesis has enabled the production of gram- and even kilogram quantities of thio- and seleno-sugar building blocks, biocatalysis has proven an effective tool for chiral resolution and the rapid development of new libraries of chalcogen-modified analogs. A synergistic approach combining chemical synthesis and biocatalysis will accelerate the development of further generations of these important materials. The inclusion of sulfur or selenium may confer desirable properties, such as lower toxicity or improved pharmacokinetics, but it is worth noting that a reduction in cytotoxic potency can often ensue. Notwithstanding this, incorporating chalcogens into a nucleoside scaffold imparts considerable value toward the pursuit of next generation structural motifs.

## Data Availability

The data underlying this study are available in the published article.

## References

[ref1] Jordheim L. P.; Durantel D.; Zoulim F.; Dumontet C. Advances in the Development of Nucleoside and Nucleotide Analogues for Cancer and Viral Diseases. Nat. Rev. Drug. Discovery 2013, 12, 447–464. 10.1038/nrd4010. 23722347

[ref2] Guinan M.; Benckendorff C.; Smith M.; Miller G. J. Recent Advances in the Chemical Synthesis and Evaluation of Anticancer Nucleoside Analogues. Molecules 2020, 25 (9), 2050 10.3390/molecules25092050. 32354007 PMC7248840

[ref3] Eastman R. T.; Roth J. S.; Brimacombe K. R.; Simeonov A.; Shen M.; Patnaik S.; Hall M. D. Remdesivir: A Review of Its Discovery and Development Leading to Emergency Use Authorization for Treatment of COVID-19. ACS. Cent. Sci. 2020, 6 (5), 672–683. 10.1021/acscentsci.0c00489. 32483554 PMC7202249

[ref4] McIntosh J. A.; Benkovics T.; Silverman S. M.; Huffman M. A.; Kong J.; Maligres P. E.; Itoh T.; Yang H.; Verma D.; Pan W.; Ho H. I.; Vroom J.; Knight A. M.; Hurtak J. A.; Klapars A.; Fryszkowska A.; Morris W. J.; Strotman N. A.; Murphy G. S.; Maloney K. M.; Fier P. S. Engineered Ribosyl-1-Kinase Enables Concise Synthesis of Molnupiravir, an Antiviral for COVID-19. ACS. Cent. Sci. 2021, 7 (12), 1980–1985. 10.1021/acscentsci.1c00608. 34963891 PMC8704035

[ref5] Ding D.; Fang Z.; Kim S. C.; O’Flaherty D. K.; Jia X.; Stone T. B.; Zhou L.; Szostak J. W. Unusual Base Pair between Two 2-Thiouridines and Its Implication for Nonenzymatic RNA Copying. J. Am. Chem. Soc. 2024, 146 (6), 3861–3871. 10.1021/jacs.3c11158. 38293747 PMC10870715

[ref6] Smith W. S.; Sierzputowska-Gracz H.; Agris P.; Sochacka E.; Malkiewicz A. Chemistry and Structure of Modified Uridine Dinucleosides Are Determined by Thiolation. J. Am. Chem. Soc. 1992, 114 (21), 7989–7997. 10.1021/ja00047a005.

[ref7] Tuorto F.; Lyko F. Genome Recoding by TRNA Modifications. Open Biol. 2016, 6 (12), 160287 10.1098/rsob.160287. 27974624 PMC5204126

[ref8] Reich H. J.; Hondal R. J. Why Nature Chose Selenium. ACS Chem. Biol. 2016, 11 (4), 821–841. 10.1021/acschembio.6b00031. 26949981

[ref9] Kulik K.; Sadowska K.; Wielgus E.; Pacholczyk-Sienicka B.; Sochacka E.; Nawrot B. 2-Selenouridine, a Modified Nucleoside of Bacterial TRNAs, Its Reactivity in the Presence of Oxidizing and Reducing Reagents. Int. J. Mol. Sci. 2022, 23 (14), 7973 10.3390/ijms23147973. 35887319 PMC9325004

[ref10] Wittwer A. J.; Stadtman T. C. Biosynthesis of 5-Methylaminomethyl-2-Selenouridine, a Naturally Occurring Nucleoside in Escherichia Coli TRNA. Arch. Biochem. Biophys. 1986, 248 (2), 540–550. 10.1016/0003-9861(86)90507-2. 2874771

[ref11] Reist E. J.; Gueffroy D. E.; Goodman L. Synthesis of 4-Thio-d- and -l-Ribofuranose and the Corresponding Adenine Nucleosides. J. Am. Chem. Soc. 1964, 86 (24), 5658–5663. 10.1021/ja01078a050.

[ref12] Dyson M. R.; Coe P. L.; Walker R. T. The Synthesis and Antiviral Activity of Some 4′-Thio-2′-Deoxy Nucleoside Analogues. J. Med. Chem. 1991, 34 (9), 2782–2786. 10.1021/jm00113a016. 1654428

[ref13] Dyson M. R.; Coe P. L.; Walker R. T. An Improved Synthesis of Benzyl 3,5-Di-O-Benzyl-2-Deoxy-1,4-Dithio-d-Erythro-Pentofuranoside, an Intermediate in the Synthesis of 4′-Thionucleosides. Carbohydr. Res. 1992, 216 (C), 237–248. 10.1016/0008-6215(92)84165-O.

[ref14] Haraguchi K.; Takahashi H.; Shiina N.; Horii C.; Yoshimura Y.; Nishikawa A.; Sasakura E.; Nakamura K. T.; Tanaka H. Stereoselective Synthesis of the β-Anomer of 4′-Thionucleosides Based on Electrophilic Glycosidation to 4-Thiofuranoid Glycals. J. Org. Chem. 2002, 67 (17), 5919–5927. 10.1021/jo020037x. 12182623

[ref15] Haraguchi K.; Shimada H.; Kimura K.; Akutsu G.; Tanaka H.; Abe H.; Hamasaki T.; Baba M.; Gullen E. A.; Dutschman G. E.; Cheng Y. C.; Balzarini J. Synthesis of 4′-Ethynyl-2′-Deoxy-4′-Thioribonucleosides and Discovery of a Highly Potent and Less Toxic NRTI. ACS Med. Chem. Lett. 2011, 2 (9), 692–697. 10.1021/ml2001054. 23795238 PMC3686520

[ref16] Secrist J. A.; Tiwari K. N.; Riordan J. M.; Montgomery J. A. Synthesis and Biological Activity of 2’-Deoxy-4’-Thio Pyrimidine Nucleosides. J. Med. Chem. 1991, 34, 2361–2366. 10.1021/jm00112a007. 1652015

[ref17] Van Draanen N. A.; Freeman G. A.; Short S. A.; Harvey R.; Jansen R.; Szczech G.; Koszalka G. W. Synthesis and Antiviral Activity of 2′-Deoxy-4′-Thio Purine Nucleosides. J. Med. Chem. 1996, 39 (2), 538–542. 10.1021/jm950701k. 8558524

[ref18] Dande P.; Prakash T. P.; Sioufi N.; Gaus H.; Jarres R.; Berdeja A.; Swayze E. E.; Griffey R. H.; Bhat B. Improving RNA Interference in Mammalian Cells by 4’-Thio-Modified Small Interfering RNA (SiRNA): Effect on SiRNA Activity and Nuclease Stability When Used in Combination with 2’-O-Alkyl Modifications. J. Med. Chem. 2006, 49 (5), 1624–1634. 10.1021/jm050822c. 16509579

[ref19] Dukhan D.; Bosc E.; Peyronnet J.; Storer R.; Gosselin G. Synthesis of 2’-C-Methyl-4’-Thio Ribonucleosides. Nucleosides Nucleotides Nucleic Acids 2005, 24 (5–7), 577–580. 10.1081/NCN-200061823. 16247991

[ref20] Jeong L. S.; Pal S.; Choe S. A.; Choi W. J.; Jacobson K. A.; Gao Z.-G.; Klutz A. M.; Hou X.; Kim H. O.; Lee H. W.; Lee S. K.; Tosh D. K.; Moon H. R. Structure-Activity Relationships of Truncated D- and l-4’-Thioadenosine Derivatives as Species-Independent A3 Adenosine Receptor Antagonists. J. Med. Chem. 2008, 51 (20), 6609–6613. 10.1021/jm8008647. 18811138 PMC3616494

[ref21] Siddiqi S. M.; Jacobson K. A.; Esker J. L.; Olah M. E.; Ji X.-D.; Melman N.; Tiwari K. N.; Secrist J. A.; Schneller S. W.; Cristalli G.; Stiles G. L.; Johnson C. R.; Ijzerman A. P. Search for New Purine-and Ribose-Modified Adenosine Analogues as Selective Agonists and Antagonists at Adenosine Receptors. J. Med. Chem. 1995, 38, 1174–1188. 10.1021/jm00007a014. 7707320 PMC3457658

[ref22] Lee H. W.; Kim H. O.; Choi W. J.; Choi S.; Lee J. H.; Park S. G.; Yoo L.; Jacobson K. A.; Jeong L. S. Design, Synthesis, and Binding of Homologated Truncated 4’-Thioadenosine Derivatives at the Human A3 Adenosine Receptors. Bioorg. Med. Chem. 2010, 18 (19), 7015–7021. 10.1016/j.bmc.2010.08.018. 20826090 PMC3724522

[ref23] Haraguchi K.; Kumamoto H.; Konno K.; Yagi H.; Tatano Y.; Odanaka Y.; Shimbara Matsubayashi S.; Snoeck R.; Andrei G. Synthesis of 4′-Substituted 2′-Deoxy-4′-Thiocytidines and Its Evaluation for Antineoplastic and Antiviral Activities. Tetrahedron 2019, 75 (33), 4542–4555. 10.1016/j.tet.2019.06.044.

[ref24] Wakamatsu H.; Nitta K.; Shoji N.; Natori Y.; Saito Y.; Yoshimura Y. Practical Synthesis of 4′-Thioribonucleosides from L-Arabinose via Novel Reductive Ring-Contraction Reaction and Pummerer-Type Thioglycosylation. Curr. Protoc. Nucleic. Acid. Chem. 2017, 71 (1), 1.43.1–1.43.12. 10.1002/cpnc.45. 29275538

[ref25] Dentmon Z. W.; Kaiser T. M.; Liotta D. C. Synthesis and Antiviral Activity of a Series of 2′-C-Methyl-4′-Thionucleoside Monophosphate Prodrugs. Molecules 2020, 25 (21), 5165 10.3390/molecules25215165. 33171951 PMC7664256

[ref26] Dukhan D.; Bosc E.; Peyronnet J.; Storer R.; Gosselin G. Synthesis of 2’-C-Methyl-4’-Thio Ribonucleosides. Nucleosides Nucleotides Nucleic Acids 2005, 24 (5–7), 577–580. 10.1081/NCN-200061823. 16247991

[ref27] Thottassery J. V.; Sambandam V.; Allan P. W.; Maddry J. A.; Maxuitenko Y.; Tiwari K.; Hollingshead M.; Parker W. B. Novel DNA Methyltransferase-1 (DNMT1) Depleting Anticancer Nucleosides, 4′-Thio-2′-Deoxycytidine and 5-Aza-4′-Thio-2′-Deoxycytidine. Cancer Chemother. Pharmacol. 2014, 74, 291–302. 10.1007/s00280-014-2503-z. 24908436 PMC4194194

[ref28] Wishka D. G.; Lopez O. D.; Rudchenko V. F.; Huang G.; Bahde R.; Kumar V.; Denysenko S. M.; Zhang L.; Zhang M.; Teicher B. A.; Morris J. The Development of β-Selective Glycosylation Reactions with Benzyl Substituted 2-Deoxy-1,4-Dithio-D-Erythro-Pentofuranosides: Enabling Practical Multi-Gram Syntheses of 4’-Thio-2’-Deoxycytidine (T-DCyd) and 5-Aza-4’-Thio-2’-Deoxycytidine (Aza-T-DCyd) to Support Clinical Development. Nucleosides Nucleotides Nucleic Acids 2021, 40 (1), 68–95. 10.1080/15257770.2020.1832694. 33063584

[ref29] Eymard C.; Manchoju A.; Almazloum A.; Dostie S.; Prévost M.; Nemer M.; Guindon Y. Synthesis of 4′-Thionucleoside Analogues Bearing a C2′ Stereogenic All-Carbon Quaternary Center. Molecules 2024, 29 (7), 1647 10.3390/molecules29071647. 38611926 PMC11013827

[ref30] Morris J.; Wishka D. G.; Lopez O. D.; Rudchenko V.; Huang G.; Hoffman S. N.; Borgel S.; Georgius K.; Carter J.; Stotler H.; Kunkel M. W.; Collins J. M.; Hollingshead M. G.; Teicher B. A. F-Aza-T-DCyd (NSC801845), a Novel Cytidine Analog, in Comparative Cell Culture and Xenograft Studies with the Clinical Candidates T-DCyd, F-T-DCyd, and Aza-T-DCyd. Mol. Cancer. Ther. 2021, 20 (4), 625–631. 10.1158/1535-7163.MCT-20-0738. 33811149 PMC8030693

[ref31] Guinan M.; Huang N.; Hawes C. S.; Lima M. A.; Smith M.; Miller G. J. Chemical Synthesis of 4′-Thio and 4′-Sulfinyl Pyrimidine Nucleoside Analogues. Org. Biomol. Chem. 2022, 20 (7), 1401–1406. 10.1039/D1OB02097H. 34806745

[ref32] Guinan M.; Huang N.; Smith M.; Miller G. J. Design, Chemical Synthesis and Antiviral Evaluation of 2′-Deoxy-2′-Fluoro-2′-C-Methyl-4′-Thionucleosides. Bioorg. Med. Chem. Lett. 2022, 61, 128605 10.1016/j.bmcl.2022.128605. 35123007

[ref33] Jeong L. S.; Tosh D. K.; Kim H. O.; Wang T.; Hou X.; Yun H. S.; Kwon Y.; Lee S. K.; Choi J.; Zhao L. X. First Synthesis of 4′-Selenonucleosides Showing Unusual Southern Conformation. Org. Lett. 2008, 10 (2), 209–212. 10.1021/ol7025558. 18088134

[ref34] Yu J.; Kim J. H.; Lee H. W.; Alexander V.; Ahn H. C.; Choi W. J.; Choi J.; Jeong L. S. New RNA Purine Building Blocks, 4′-Selenopurine Nucleosides: First Synthesis and Unusual Mixture of Sugar Puckerings. Chem.—Eur. J. 2013, 19 (18), 5528–5532. 10.1002/chem.201300741. 23553943

[ref35] Kim J. H.; Yu J.; Alexander V.; Choi J. H.; Song J.; Lee H. W.; Kim H. O.; Choi J.; Lee S. K.; Jeong L. S. Structure-Activity Relationships of 2′-Modified-4′- Selenoarabinofuranosyl-Pyrimidines as Anticancer Agents. Eur. J. Med. Chem. 2014, 83, 208–225. 10.1016/j.ejmech.2014.06.031. 24956556

[ref36] Lee H.; Jarhad D. B.; Yu J.; Lee C.; Jeong L. S. Asymmetric Synthesis of 2′- C-Methyl-4′-Selenonucleosides as Anti-Hepatitis C Virus Agents. J. Org. Chem. 2019, 84 (22), 14414–14426. 10.1021/acs.joc.9b01462. 31608633

[ref37] Lee H.; Jarhad D. B.; Lee A.; Lee C.; Jeong L. S. 4′-Selenonucleosides: Regio- and Stereoselective Synthesis of Novel Ribavirin and Acadesine Analogs as Anti-Hepatitis C Virus (HCV) Agents. Asian J. Org. Chem. 2021, 10 (11), 2993–2999. 10.1002/ajoc.202100563.

[ref38] Saito-Tarashima N.; Ota M.; Minakawa N. Synthesis of 4′-Selenoribonucleosides, the Building Blocks of 4′-SelenoRNA, Using a Hypervalent Iodine. Curr. Protoc. Nucleic. Acid. Chem. 2017, 70 (1), 1.40.1–1.40.21. 10.1002/cpnc.34. 28921498

[ref39] Sahu P. K.; Kim G.; Yu J.; Ahn J. Y.; Song J.; Choi Y.; Jin X.; Kim J. H.; Lee S. K.; Park S.; Jeong L. S. Stereoselective Synthesis of 4′-Selenonucleosides via Seleno-Michael Reaction as Potent Antiviral Agents. Org. Lett. 2014, 16 (21), 5796–5799. 10.1021/ol502899b. 25340622

[ref40] Kim G.; Choi Y.; Sahu P. K.; Yu J.; Qu S.; Lee D.; Jeong L. S. Stereoselective Synthesis of d −5-Homo-4-Selenoribose as a Versatile Intermediate for 4′-Selenonucleosides. Org. Lett. 2015, 17 (18), 4636–4639. 10.1021/acs.orglett.5b02393. 26348005

[ref41] Qu S.; Kim G.; Yu J.; Sahu P. K.; Choi Y.; Naik S. D.; Jeong L. S. Synthesis and Anti-HIV Activity of 5′-Homo-2′,3′-dideoxy-2′,3′-didehydro-4′-selenonucleosides (5′-Homo-4′-Se-d4 Ns). Asian J. Org. Chem. 2016, 5 (6), 735–741. 10.1002/ajoc.201600154.

[ref42] Singleton J.; Sahteli K.; Hoberg J. O. Synthesis of 2,3-Dihydroxyhex-4-enoates by Palladium-Catalyzed Allylic Alkylations of Carbohydrate Vinyl Lactones. Synthesis 2008, 2008 (22), 3682–3686. 10.1055/s-0028-1083203.

[ref43] Jayakanthan K.; Johnston B. D.; Pinto B. M. Stereoselective Synthesis of 4′-Selenonucleosides Using the Pummerer Glycosylation Reaction. Carbohydr. Res. 2008, 343 (10–11), 1790–1800. 10.1016/j.carres.2008.02.014. 18316068

[ref44] Ishii K.; Saito-Tarashima N.; Ota M.; Yamamoto S.; Okamoto Y.; Tanaka Y.; Minakawa N. Practical Synthesis of 4′-Selenopurine Nucleosides by Combining Chlorinated Purines and ‘Armed’ 4-Selenosugar. Tetrahedron 2016, 72 (41), 6589–6594. 10.1016/j.tet.2016.08.071.

[ref45] Jeong L. S.; Tosh D. K.; Choi W. J.; Lee S. K.; Kang Y. J.; Choi S.; Lee J. H.; Lee H.; Lee H. W.; Kim H. O. Discovery of a New Template for Anticancer Agents: 2′-Deoxy-2′- Fluoro-4′-Selenoarabinofuranosyl-Cytosine (2′-F-4′-Seleno-Ara- C). J. Med. Chem. 2009, 52 (17), 5303–5306. 10.1021/jm900852b. 19691349

[ref46] Yu J.; Sahu P. K.; Kim G.; Qu S.; Choi Y.; Song J.; Lee S. K.; Noh M.; Park S.; Jeong L. S. Design, Synthesis and Cellular Metabolism Study of 4′-Selenonucleosides. Future Med. Chem. 2015, 7 (13), 1643–1655. 10.4155/fmc.15.102. 26399780

[ref47] Sahu P. K.; Naik S. D.; Yu J.; Jeong L. S. 4′-Selenonucleosides as Next-Generation Nucleosides. Eur. J. Org. Chem. 2015, 2015 (28), 6115–6124. 10.1002/ejoc.201500429.

[ref48] Migliaccio G.; Tomassini J. E.; Carroll S. S.; Tomei L.; Altamura S.; Bhat B.; Bartholomew L.; Bosserman M. R.; Ceccacci A.; Colwell L. F.; Cortese R.; De Francesco R.; Eldrup A. B.; Getty K. L.; Hou X. S.; LaFemina R. L.; Ludmerer S. W.; MacCoss M.; McMasters D. R.; Stahlhut M. W.; Olsen D. B.; Hazuda D. J.; Flores O. A. Characterization of Resistance to Non-Obligate Chain-Terminating Ribonucleoside Analogs That Inhibit Hepatitis C Virus Replication in Vitro. J. Biol. Chem. 2003, 278 (49), 49164–49170. 10.1074/jbc.M305041200. 12966103

[ref49] Chu C. K.; Ma L.; Olgen S.; Pierra C.; Du J.; Gumina G.; Gullen E.; Cheng Y. C.; Schinazi R. F. Synthesis and Antiviral Activity of Oxaselenolane Nucleosides. J. Med. Chem. 2000, 43 (21), 3906–3912. 10.1021/jm990113x. 11052795

[ref50] Kim H. O.; Schinazi R. F.; Shanmuganathan K.; Jeong L. S.; Beach J. W.; Nampalli S.; Cannon D. L.; Chu C. K. L-β-(2S,4S)- and L-α-(2S,4R)-Dioxolanyl Nucleosides as Potential Anti-HIV Agents: Asymmetric Synthesis and Structure-Activity Relationships. J. Med. Chem. 1993, 36 (5), 519–528. 10.1021/jm00057a001. 8496934

[ref51] Mandala D.; Chada S.; Watts P. Semi-Continuous Multi-Step Synthesis of Lamivudine. Org. Biomol. Chem. 2017, 15 (16), 3444–3454. 10.1039/C7OB00480J. 28362445

[ref52] Caso M. F.; D’Alonzo D.; D’Errico S.; Palumbo G.; Guaragna A. Highly Stereoselective Synthesis of Lamivudine (3TC) and Emtricitabine (FTC) by a Novel N -Glycosidation Procedure. Org. Lett. 2015, 17 (11), 2626–2629. 10.1021/acs.orglett.5b00982. 25965958

[ref53] Mandala D.; Watts P. An Improved Synthesis of Lamivudine and Emtricitabine. ChemistrySelect 2017, 2 (3), 1102–1105. 10.1002/slct.201700052.

[ref54] Kashinath K.; Snead D. R.; Burns J. M.; Stringham R. W.; Gupton B. F.; McQuade D. T. Synthesis of an Oxathiolane Drug Substance Intermediate Guided by Constraint-Driven Innovation. Org. Process Res. Dev. 2020, 24 (10), 2266–2270. 10.1021/acs.oprd.0c00145. 33100812 PMC7574620

[ref55] De Souza J. M.; Berton M.; Snead D. R.; McQuade D. T. A Continuous Flow Sulfuryl Chloride-Based Reaction - Synthesis of a Key Intermediate in a New Route toward Emtricitabine and Lamivudine. Org. Process Res. Dev. 2020, 24 (10), 2271–2280. 10.1021/acs.oprd.0c00146. 33100813 PMC7574626

[ref56] Aher U. P.; Srivastava D.; Jadhav H. S.; Singh G. P.; Jayashree B. S.; Shenoy G. G. Large-Scale Stereoselective Synthesis of 1,3-Oxathiolane Nucleoside, Lamivudine, via ZrCl4-Mediated N-Glycosylation. Org. Process Res. Dev. 2020, 24 (3), 387–397. 10.1021/acs.oprd.9b00414.

[ref57] Hu L.; Schaufelberger F.; Zhang Y.; Ramström O. Efficient Asymmetric Synthesis of Lamivudine via Enzymatic Dynamic Kinetic Resolution. Chem. Commun. 2013, 49 (88), 10376–10378. 10.1039/C3CC45551C. 24071972

[ref58] Ren Y.; Hu L.; Ramström O. Multienzymatic Cascade Synthesis of an Enantiopure (2R,5R)-1,3-Oxathiolane Anti-HIV Agent Precursor. Mol. Catal. 2019, 468, 52–56. 10.1016/j.mcat.2019.02.013.

[ref59] Chen Y.; Zhang X.; Zheng G.; Gao S. Preparation of the Enantiomerically Enriched Precursor of Lamivudine (3TC^TM^) via Asymmetric Catalysis Mediated by Klebsiella Oxytoca. Process Biochem. 2019, 81, 77–84. 10.1016/j.procbio.2019.03.025.

[ref60] Zhang Y.; Sun Y.; Tang H.; Zhao Q.; Ren W.; Lv K.; Yang F.; Wang F.; Liu J. One-Pot Enzymatic Synthesis of Enantiopure 1,3-Oxathiolanes Using Trichosporon Laibachii Lipase and the Kinetic Model. Org. Process Res. Dev. 2020, 24 (4), 579–587. 10.1021/acs.oprd.0c00010.

[ref61] Gunaga P.; Baba M.; Jeong L. S. Asymmetric Synthesis of Novel Thioiso Dideoxynucleosides with Exocyclic Methylene as Potential Antiviral Agents. J. Org. Chem. 2004, 69 (9), 3208–3211. 10.1021/jo035735b. 15104467

[ref62] Moon H. R.; Kim H. O.; Lee S. K.; Choi W. J.; Chun M. W.; Jeong L. S. Synthesis and Biological Evaluation of Novel Thioapio Dideoxynucleosides. Bioorg. Med. Chem. 2002, 10 (5), 1499–1507. 10.1016/S0968-0896(01)00417-5. 11886812

[ref63] Jeong L. S.; Kim H. O.; Moon H. R.; Hong J. H.; Yoo S. J.; Choi W. J.; Chun M. W.; Lee C.-K. Syntheses and Structure-Activity Relationships of Novel Apio and Thioapio Dideoxydidehydronucleosides as Anti-HCMV Agents. J. Med. Chem. 2001, 44 (5), 806–813. 10.1021/jm000342f. 11262090

[ref64] Huang K. X.; Xie M. S.; Sang J. W.; Qu G. R.; Guo H. M. Asymmetric Synthesis of 3-Amine-Tetrahydrothiophenes with a Quaternary Stereocenter via Nickel(II)/Trisoxazoline-Catalyzed Sulfa-Michael/Aldol Cascade Reaction: Divergent Access to Chiral Thionucleosides. Org. Lett. 2021, 23 (1), 81–86. 10.1021/acs.orglett.0c03747. 33332122

[ref65] Hao E. J.; Zhao Y.; Yu M.; Li X. J.; Wang K. X.; Su F. Y.; Liang Y. R.; Wang Y.; Guo H. M. Discovery, Synthesis, and Activity Evaluation of Novel Five-Membered Sulfur-Containing Heterocyclic Nucleosides as Potential Anticancer Agents In Vitro and In Vivo. J. Med. Chem. 2024, 67, 12553–12570. 10.1021/acs.jmedchem.4c00443. 39016216

[ref66] Botha F.; Slavíčková M.; Pohl R.; Hocek M. Copper-Mediated Arylsulfanylations and Arylselanylations of Pyrimidine or 7-Deazapurine Nucleosides and Nucleotides. Org. Biomol. Chem. 2016, 14 (42), 10018–10022. 10.1039/C6OB01917J. 27722411

[ref67] Zhou X.; Yan W.; Zhang C.; Yang Z.; Neubauer P.; Mikhailopulo I. A.; Huang Z. Biocatalytic Synthesis of Seleno-, Thio- and Chloro-Nucleobase Modified Nucleosides by Thermostable Nucleoside Phosphorylases. Catal. Commun. 2019, 121, 32–37. 10.1016/j.catcom.2018.12.004.

[ref68] Hellendahl K. F.; Kaspar F.; Zhou X.; Yang Z.; Huang Z.; Neubauer P.; Kurreck A. Optimized Biocatalytic Synthesis of 2-Selenopyrimidine Nucleosides by Transglycosylation. ChemBioChem. 2021, 22 (11), 2002–2009. 10.1002/cbic.202100067. 33594780 PMC8251958

[ref69] Westarp S.; Benckendorff C. M. M.; Motter J.; Röhrs V.; Sanghvi Y. S.; Neubauer P.; Kurreck J.; Kurreck A.; Miller G. J. Biocatalytic Nucleobase Diversification of 4′-Thionucleosides and Application of Derived 5-Ethynyl-4′-Thiouridine for RNA Synthesis Detection. Angew. Chem., Int. Ed. 2024, 63, e202405040 10.1002/anie.202405040. 38785103

